# An art style classification network integrating contrastive learning and counterfactual attention

**DOI:** 10.1371/journal.pone.0343954

**Published:** 2026-06-26

**Authors:** Meng Wang, Fan Xia, Ting Yang, Yi Zou, Yang Zhou

**Affiliations:** School of Art and Media, Jincheng College of Nanjing University of Aeronautics and Astronautics, Nanjing, China; PLOS, UNITED KINGDOM OF GREAT BRITAIN AND NORTHERN IRELAND

## Abstract

Art style classification is a fundamental task for digital art analysis and intelligent cultural heritage management. However, existing methods often fail to deliver stable and interpretable results due to dispersed style cues, complex local textures, strong interference from background and subject content, and the difficulty of identifying regions that are causally relevant to style recognition. To address these issues, we propose a Multi Source Collaborative Style Network, termed MCS-Net, which jointly models global composition and local brushstroke textures within a unified framework. MCS-Net consists of four modules, namely a basic style feature encoding module, an attention generation module, a style contrastive learning module, and a counterfactual attention module. The attention generation module discovers multiple complementary discriminative regions. The contrastive learning module constructs a more discriminative embedding space to improve the separability of fine-grained styles. The counterfactual attention module builds counterfactual attention samples to explicitly estimate the true contribution of regions to predictions, thereby suppressing spurious activations, improving robustness, and providing counterfactual-supported evidence for model interpretation. Experiments on three public datasets, WikiArt, MultitaskPainting100k, and Pandora18k, demonstrate that MCS-Net outperforms representative baselines across standard evaluation metrics.

## 1. Introduction

Art style classification is an important research direction at the intersection of art history and computer vision, and it has long held broad academic and practical value [1,2]. As society increasingly values spiritual and cultural well-being, visual art forms such as painting, architecture, and photography have become deeply integrated into everyday life, serving as major carriers of cultural expression and aesthetic experience [3]. The visual language of artworks contains rich historical information and cultural imagery, and different artistic styles reflect the social thought, philosophical ideas, and aesthetic tendencies of specific periods [4]. With the advent of the digital era, artworks have been collected, stored, and disseminated at scale in the form of images, enabling people to appreciate artworks from different times and regions through online platforms and virtual exhibitions. However, how to automatically recognize and analyze the styles of these art images using computer vision and artificial intelligence has become a central concern for both academia and industry [5,6].

Automated art style classification is not only theoretically meaningful but also has substantial practical potential. First, it can help non-expert audiences understand differences among art movements, thereby promoting public art literacy and the popularization of art education [7]. Second, style recognition models can provide intelligent support for online art platforms, digital museums, and personalized recommendation systems, improving user interaction and viewing experiences [8]. In addition, art style classification is highly valuable for art provenance, cultural heritage preservation, artwork authentication, and art historical research [9]. By analyzing visual features of artworks in depth, computational methods can reveal patterns in the evolution of artistic styles and explore artists’ intentions as well as internal links among art schools, offering new technical pathways for understanding the dissemination of art culture and the development of visual language.

However, automated art style recognition remains a complex and challenging task [10,11]. This challenge first stems from the diversity and abstract nature of artworks. Artistic style is expressed not only through surface visual elements such as color, line, texture, and composition, but also through deeper aspects including an artist’s emotional expression, conceptual imagery, and cultural background. These complex characteristics that span both form and semantics make it difficult for machine learning models to accurately capture and interpret the essence of style. In addition, the boundaries between different art movements are often ambiguous, and style fusion frequently occurs. Substantial individualized variations also exist within the same movement, which makes it difficult for conventional classification algorithms to establish clear decision boundaries. The problem is further compounded by the fact that art image annotations often rely on expert judgment and are subjective, which can introduce label noise and increase learning difficulty. Moreover, differences in artistic medium, illumination, texture, and preservation conditions can lead to inconsistent feature distributions, thereby affecting training stability and generalization performance.

Early studies on art style recognition largely relied on handcrafted visual features and traditional machine learning algorithms. Researchers typically extracted features using color histograms, local binary patterns, scale-invariant feature transform, or gist descriptors, and then performed classification with support vector machines, k-nearest neighbors, or random forests. By quantitatively analyzing color, texture, edges, and brushstrokes, these methods partially revealed differences among artistic styles. For example, Nunez-Garcia et al. [12] combined Gabor filters and local binary patterns for painting genre classification, and further compared the performance of multiple color and texture features for art style recognition [13]. Kumar et al. [14] validated the effectiveness of traditional approaches based on local binary patterns and Gabor features for Indian traditional art image classification. Beyond low-level visual descriptors, some studies attempted to extract higher-level features such as compositional structure, spatial layout, and the distribution of artistic elements to compensate for the limited ability of handcrafted features to model abstract semantics [15–17]. Nevertheless, these traditional methods depend heavily on expert experience and feature engineering, adapt poorly to diverse styles, perform limitedly on complex art images, and lack the capacity to model deeper semantic aspects of artistic style.

With the rise of deep learning, convolutional neural networks have gradually replaced traditional methods and become the dominant approach for art image style classification. Convolutional neural networks can automatically learn multi-level representations, ranging from low-level lines and colors to high-level semantics and affect, demonstrating strong capability in modeling complex images. Sergey et al. [18] were among the first to apply deep learning to art style recognition and proposed a method that uses convolutional features for multi-dimensional style classification. Elgammal et al. [19] explored the potential of convolutional neural networks in artwork analysis and revealed intrinsic connections between model predictions and the art-historical context. Lecoutre et al. [20] employed deep residual networks to recognize painting styles and substantially improved accuracy. Subsequently, research expanded toward cross-task and multi-modal directions. Hicsonmez et al. [21] used convolutional neural networks to classify children’s illustration styles, demonstrating the expressive power of deep features for abstract style classification. Xiao et al. [22] achieved hierarchical style modeling through multi-scale feature fusion. Imran et al. [23] proposed a hybrid neural network that captures both fine-grained brushstroke characteristics and overall compositional relations. Luo et al. [24] designed a lightweight model based on self-supervised learning and dual-teacher distillation, achieving strong performance on the Pandora18k and WikiArt datasets. Wang [25] introduced a multi-level feature compression mechanism to reduce storage and computation while maintaining high accuracy. These results indicate that deep neural networks have clear advantages in capturing the complex semantic characteristics of artworks and have opened new directions for art style recognition.

In recent years, the introduction of Transformer architectures has brought new breakthroughs to art style classification. Unlike convolutional neural networks, Transformers are based on self-attention and can capture global dependencies during feature modeling, providing stronger capacity for understanding overall structure and semantics in images. Vision Transformer segments an art image into fixed-size patches and computes correlations among local regions via self-attention, enabling unified modeling of global features. This design mitigates the limitation of convolutional receptive fields and allows the model to capture cross-region visual semantic relations in artworks. Recently, researchers [26,27] have explored combinations of convolutional neural networks and Transformers to leverage both local detail modeling and global structural understanding. In addition, İnal and Çiftçi [28] systematically compared several CNN and Vision Transformer models on the WikiArt dataset and showed that Transformer-based methods generally outperformed CNN-based methods in art painting classification, further highlighting the effectiveness of global dependency modeling for style recognition. For example, Zhou et al. [29] proposed a multi-scale dilated convolution Transformer that fuses convolutional feature extraction with self-attention and significantly improves performance in art style recognition. Yin [30] proposed an efficient Transformer design and improved network stability and feature extraction by introducing E-Attention and dilated convolution modules. Liu et al. [31] designed a soft-threshold attention mechanism to optimize channel selection and strengthen the focus on key visual regions in art images. With continued structural innovations, hybrid models that combine convolutional neural networks and Transformers have become a major trend in art style classification [32,33].

Although recent CNN-, Transformer-, and attention-based methods have improved art style classification, most existing studies still focus primarily on either global representation learning, local attention enhancement, or stronger backbone design. In contrast, artistic style is often expressed through distributed and heterogeneous cues, including local brushstroke textures, regional color transitions, and global compositional organization. Moreover, even when attention mechanisms are introduced, the attended regions are usually selected according to statistical saliency rather than their actual contribution to style discrimination. As a result, existing models may still suffer from insufficient integration of multi-region evidence, weak separability among visually similar styles, and limited support for interpretation of model decisions.

To address these issues, we propose a Multi-Source Collaborative Style Network (MCS-Net) for art style classification. Instead of relying on a single global representation or a single salient region, MCS-Net jointly models global style structure and multiple complementary local style regions within a unified framework. Furthermore, a style contrastive learning strategy is introduced to enhance the discriminability of style embeddings, and a counterfactual attention mechanism is incorporated to evaluate the contribution of attended regions through intervention-based comparison. In this way, the proposed method combines multi-region style mining, discriminative embedding learning, and counterfactual attention refinement in a coordinated manner, providing a more robust solution for complex art style recognition. The main contributions of this work are as follows:

- We propose MCS-Net, a unified framework for art style classification that jointly models global compositional structure and multiple complementary local style regions, aiming to better capture spatially distributed and heterogeneous style cues.
- We enhance style discrimination by combining multi-region representation learning with style contrastive learning and by refining attended regions with a counterfactual attention mechanism, which together improve feature separability and reduce spurious responses.
- Extensive experiments on three public datasets demonstrate that the proposed method achieves consistent performance gains over representative baseline methods, while ablation and visualization results further support the effectiveness of the proposed design.

## 2. Proposed method

### 2.1. Overview

**Fig 1:**
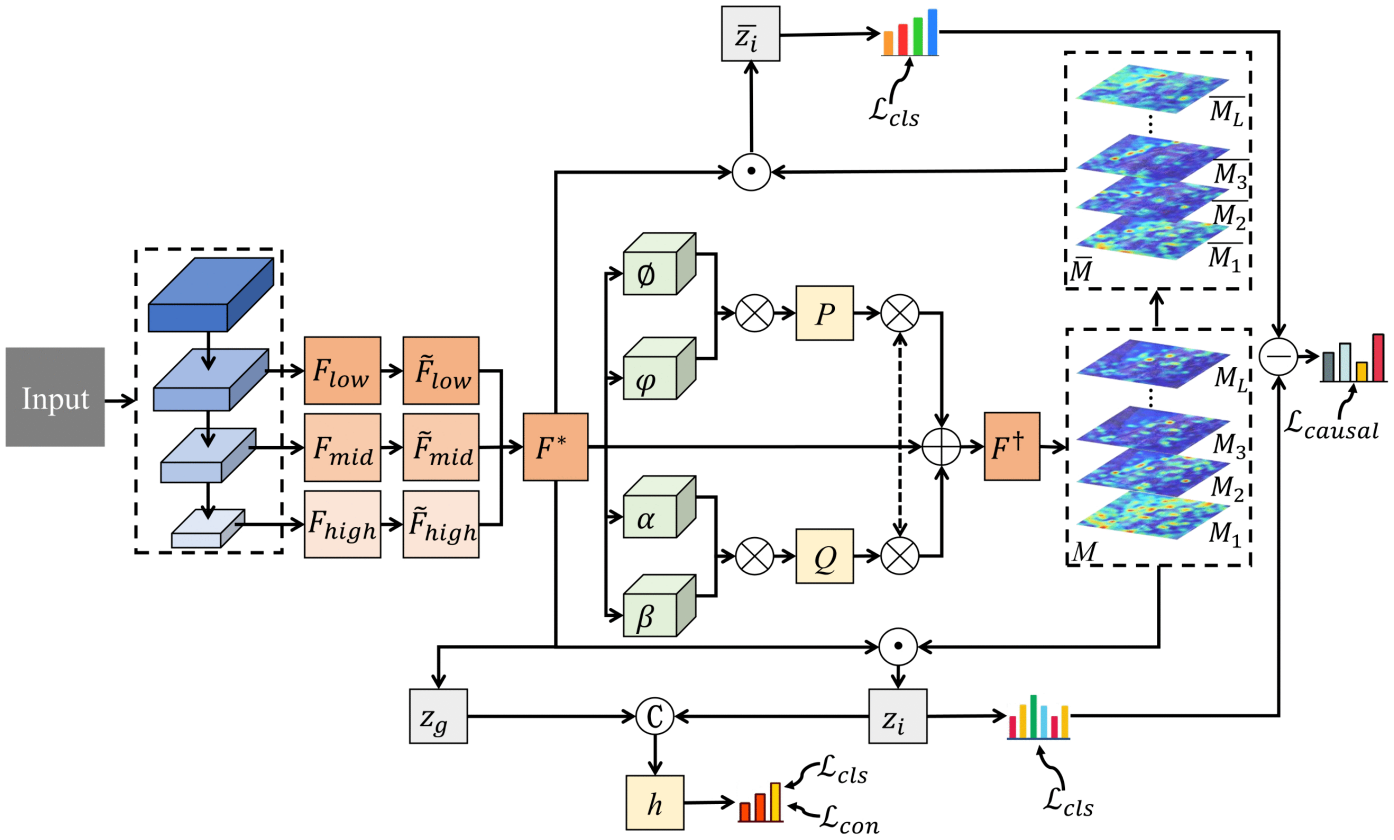
Architecture of the MCS-Net model.

To address the challenges in art style classification, including dispersed style elements, complex local textures, strong background interference, and the difficulty of identifying causal factors of style, we design a Multi-Source Collaborative Style Network, termed MCS-Net. As shown in Fig 1, MCS-Net consists of four core components: a basic style feature encoding module, an attention generation module, a style contrastive learning module, and a causal counterfactual reasoning module. These modules operate collaboratively within a unified framework to jointly model multi-level style information, ranging from global composition to local brushstrokes, and from feature similarity to style causality. First, the basic style feature encoding module performs multi-scale structural representation learning on the input image to obtain global style features that capture brushstroke textures, color distributions, and compositional layouts. Next, the attention generation module builds a region-correlation representation based on the encoded features and adaptively highlights local regions that are more likely to carry style information. On this basis, the style contrastive learning module jointly encodes global features and local region features, and applies constraints between positive and negative samples to form a clearer clustering structure of style features in the feature space. Finally, to suppress interference factors that are irrelevant to style, such as background or subject content, the causal counterfactual reasoning module constructs counterfactual attention samples to explicitly measure the true contribution of different regions to style prediction, thereby emphasizing core style factors and improving semantic stability.

### 2.2. Basic style feature encoding module

Artistic style is often expressed across multiple visual levels, including local brushstroke patterns, texture details, color composition, and overall spatial layout. These characteristics are highly non-uniformly distributed in space and jointly form style semantics across scales. Therefore, to construct a stable representation space with strong style discriminability, we first employ a multi-scale hierarchical encoding module to learn comprehensive structural style representations from the input image.

The module takes a normalized image as input and extracts multi-scale representations from low-level textures to high-level semantics through a set of progressively stacked convolutional encoding blocks. Each block consists of a convolution operator, a normalization layer, and a nonlinear activation function. The convolution kernel sizes are adjusted across layers to capture fine-grained brushstroke structures (such as stroke direction and texture granularity) as well as large-scale style layouts (such as color distribution and compositional rhythm). To ensure stable propagation of deep features, we adopt a cross-layer residual structure, which makes the abstraction of style semantics in higher layers more stable during training. Let the encoding network be ℰ(·); the basic feature map is defined as follows:


$$\begin{array}{c} F=ℰ(I),\;F\in {\mathbb{R}}^{H\times W\times C} \end{array}$$ (1)

where *H*, *W* and *C* denote the spatial dimensions and the channel number of the feature map, respectively. This representation covers multi-level information ranging from local physical textures to abstract style patterns.

To improve the model’s sensitivity to irregular details in artworks, such as texture granules formed by material stacking, directional variations induced by brush movements, and non-uniformity caused by color transitions, we further introduce a structure-preserving perturbation augmentation strategy at the encoding stage. This strategy exposes the encoder to the natural variability of style during training. Specifically, we apply lightweight affine perturbations to the feature map, including small rotations, scaling, and shearing, together with low-energy texture noise, to simulate unavoidable visual shifts and texture fluctuations in real artistic creation. The augmented feature is defined as follows:


$$\begin{array}{c} \tilde{F}=𝒯(F)=𝒜(F)+\gamma \cdot 𝒩(F) \end{array}$$ (2)

where 𝒜(·) denotes an affine transformation that preserves local relative structure, 𝒩(·) denotes a texture-noise perturbation, and γ controls the noise intensity. This augmentation enhances the encoder’s ability to perceive brushstroke-level style cues without destroying the global composition.

In addition, considering the complementary roles of multi-scale features for style representation, we introduce a cross-layer fusion mechanism at the output stage. We aggregate features from shallow, middle, and deep layers so that the resulting integrated style representation is both detail-sensitive and structurally informative. The fusion process is formulated as follows:


$$\begin{array}{c} F^{*}=ℱ\;\left({\tilde{F}}_{low},\;{\tilde{F}}_{mid},\;{\tilde{F}}_{high}\right) \end{array}$$ (3)

where ℱ(·) denotes the cross-scale feature integration function, and $F^{*}$ is the final output style feature.

### 2.3. Attention generation module

Artistic style is typically formed by multiple spatial regions. Different regions may carry different style semantics. For example, regions with large variations in brushstroke density often contain texture-related style information, whereas regions with strong color jumps may reflect palette characteristics. Therefore, relying only on global features of the whole image is insufficient to capture these spatially heterogeneous style factors. The model should be able to adaptively identify and enhance key style regions. We design an attention generation module to mine potentially high-contribution style regions from the encoded features and to produce explicit regional representations for subsequent style contrastive learning and causal counterfactual reasoning.

This module is built on the multi-scale style features $F^{*}$ obtained in the previous stage. It models inter-region style correlations from two perspectives, namely channel dependency and spatial dependency. In the channel dimension, different channels often correspond to different style structures, such as edges, texture directionality, and color gradients. Therefore, inter-channel affinity reflects how style components are combined. We first compress features along the spatial dimension into a channel activation sequence, and then construct a channel correlation matrix through two nonlinear mapping functions so that the model can capture interactions among different style components. The formulation is as follows:


$$\begin{array}{c} P=\sigma \;\left(\phi (F^{*})\right)\cdot \sigma \;{\left(\varphi (F^{*})\right)}^{⊤} \end{array}$$ (4)

where ϕ(·) and φ(·) are channel projection functions, and σ(·) is a nonlinear activation function. Each element of *P* characterizes the style closeness between two channels, thereby encouraging the network to enhance channel combinations that are more discriminative for style recognition.

In the spatial dimension, style regions in artworks often exhibit complex contextual dependencies. For example, certain brushstroke textures may form a style pattern together with adjacent color blocks. We further construct a spatial correlation matrix by applying bilinear mappings over spatial positions of the feature map, enabling the model to capture style coherence across different locations. The formulation is as follows:


$$\begin{array}{c} Q=\tanh\;\left(\alpha (F^{*})\right)\cdot \tanh\;{\left(\beta (F^{*})\right)}^{⊤} \end{array}$$ (5)

where α(·) and β(·) are spatial projection functions. The matrix *Q* effectively describes the consistency of style responses across regions, allowing the network to recognize structural relations among style regions.

After integrating channel and spatial dependencies, the attention generation module obtains the candidate regional response via a weighted combination:


$$\begin{array}{c} F^{†}=F^{*}+\lambda_{c}\left(F^{*}P\right)+\lambda_{s}\left(QF^{*}\right) \end{array}$$ (6)

where $\lambda_{c}$ and $\lambda_{s}$ control the integration strength of channel dependency and spatial dependency, respectively. Finally, the module applies nonlinear enhancement to the fused features through a generation subnetwork and outputs a set of style-region attention maps.


$$\begin{array}{c} M=𝒢(F^{†}),\;M\in {\mathbb{R}}^{L\times H\times W} \end{array}$$ (7)

where 𝒢(·) denotes the attention-map generation function, and *L* is the number of attention maps.

To avoid redundant region representations caused by overlapping attention focus during training, we impose a region distribution constraint to regularize multiple attention maps so that they learn complementary style patterns in the image space. This regularization encourages different attention maps to maintain a moderate distinction in their focus locations, thereby improving the coverage and diversity of the overall style representation.

### 2.4. Style contrastive learning module

In artistic style recognition, relying only on attention regions at a single scale or only on global compositional features is insufficient to achieve strong style separability. Local regions contain fine-grained textures and brushstroke cues, but their limited coverage often fails to reflect the overall stylistic structure; in contrast, global features capture macro-level tone but are easily disturbed by subject shapes or background content. Therefore, we need a method that can integrate local style features and global style semantics while actively shaping a discriminative structure in the feature space. To this end, we construct a style contrastive learning module. We jointly encode the local style-region representations from the attention generation module and the global style representation from the basic style feature encoding module, and we use contrastive learning to enhance feature separation among different styles.

First, for the *L* region attention maps obtained by the attention generation module, we apply them to the basic style feature one by one to extract local representations with salient style meaning. Specifically, we obtain the *i*-th local style feature through point-wise multiplication:


$$\begin{array}{c} F_{i}=F^{*}⊙M_{i} \end{array}$$ (8)

To make these region features usable for style-level representation learning, we perform global aggregation on each local region:


$$\begin{array}{c} z_{i}=Pool(F_{i}),\;i=1,2,\cdots ,L \end{array}$$ (9)

where Pool(·) is the spatial aggregation function used to extract an overall style descriptor of the region. Meanwhile, we keep the global pooled representation of the encoded feature $F^{*}$:


$$\begin{array}{c} z_{g}=Pool(F^{*}) \end{array}$$ (10)

This global feature reflects the macro-level stylistic properties of the whole image, such as color tone and compositional rhythm.

To construct a more complete style representation, we concatenate the global feature and the local features and then apply normalization to obtain an image-level style embedding:


$$\begin{array}{c} h=Normalize([z_{g}||z_{1}||\cdots ||z_{L}]) \end{array}$$ (11)

This joint embedding contains both macro-level style structure and local style components, providing a multi-granularity basis for distinguishing different styles in the feature space.

On this basis, to further strengthen the style decision boundary, we adopt a contrastive constraint defined by the relationship between positive and negative samples. This encourages embeddings from the same style category to be more compact and embeddings from different style categories to be more dispersed. For an arbitrary image embedding *h*, let the positive sample embedding of the same style be *h*^+^, and let the embeddings of several negative samples from different styles be $h_{j}^{-}$. The contrastive loss is defined as follows:


$$\begin{array}{c} {ℒ}_{con}=-\log\frac{\exp(sim(h,h^{+})/\tau )}{\exp(sim(h,h^{+})/\tau )+\sum_{j}\exp(sim(h,h_{j}^{-})/\tau )} \end{array}$$ (12)

where sim(·) is the feature similarity function, and τ is the temperature parameter.

With the above constraint, the style embedding space forms a structured distribution: images from the same style naturally aggregate in the embedding space, while images from different styles tend to be spatially separated. This structured feature space not only improves the discriminability of the classifier, but also provides a clearer and more stable stylistic semantic basis for the subsequent causal counterfactual reasoning module.

### 2.5. Causal counterfactual attention module

In the style contrastive learning module, we have already built a discriminative style representation based on attention-guided local features and their aggregated embeddings. However, discriminative features can still be driven by statistical correlations, meaning that some regions may show strong responses in the feature space but do not necessarily play a decisive role in style discrimination. To further quantify the true contribution of different attention regions to style classification, we introduce a causal counterfactual attention mechanism. By explicitly constructing alternative attention and comparing prediction differences, we perform a causal evaluation of region-level style representations.

For the *i*-th attention region, its attention map is $M_{i}$, which yields the corresponding region style representation $z_{i}$. To construct a counterfactual setting, we intervene on the original attention distribution while keeping the backbone feature unchanged, generating a counterfactual attention map ${\bar{M}}_{i}$. Specifically, we first randomly shuffle the spatial positions of $M_{i}$ so that its high-response areas are detached from the original spatial structure, and then normalize the shuffled attention to keep its overall energy consistent with the original attention. This process is formalized as:


$$\begin{array}{c} {\bar{M}}_{i}=Normalize(Shuffle(M_{i})) \end{array}$$ (13)

where Shuffle(·) denotes a random spatial permutation of the attention map, and Normalize(·) is a normalization function used to keep the counterfactual attention on the same numerical scale. With this construction, the counterfactual attention is comparable to the original one in intensity distribution, but it no longer carries the original spatial semantic structure.

Under the counterfactual attention, the counterfactual style representation of the region is ${\bar{z}}_{i}$. By comparing the predictions under factual attention and counterfactual attention, we characterize the causal role of the region in style discrimination. The causal contribution of region *i* is defined as:


$$\begin{array}{c} \Delta_{i}=f(z_{i})-f({\bar{z}}_{i}) \end{array}$$ (14)

where f(·) is the classifier output function, and $\Delta_{i}$ measures the change in style prediction between the factual and counterfactual conditions for the same region. When a region contains visual cues that are critical for style discrimination, replacing its attention with a counterfactual one will lead to a clear prediction change, producing a large causal effect.

To strengthen the model’s reliance on causally critical regions during training, we introduce a constraint term based on causal contribution to optimize attention-guided style representations. This constraint encourages larger absolute causal effects across regions, so the model gradually increases attention to regions that are sensitive to counterfactual replacement. It is defined as:


$$\begin{array}{c} {ℒ}_{causal}=-\sum_{i=1}^{L}\left|\Delta_{i}\right| \end{array}$$ (15)

With this causal intervention training, the attention distribution is no longer driven only by correlation, but progressively concentrates on regions that remain decisive under counterfactual conditions. As a result, the model can effectively suppress spurious responses introduced by background structure or other non-style factors, improving the stability and reliability of artistic style classification in complex visual scenes.

### 2.6. Model training strategy

To achieve collaborative optimization of the four modules, we adopt an end-to-end joint training scheme. In the overall framework, classification prediction serves as the primary supervision signal and is directly applied to the final representation jointly formed by global features and regional features. The classification loss uses the standard cross-entropy form:


$$\begin{array}{c} {ℒ}_{cls}=-\sum_{k=1}^{K}y_{k}\log({\hat{y}}_{k}) \end{array}$$ (16)

where $y_{k}$ is the one-hot encoding of the ground-truth style label, and ${\hat{y}}_{k}$ is the predicted probability for the *k*-th style class. This loss ensures that the model learns accurate style discrimination from a global perspective.

In addition to the classification loss, the style contrastive learning module provides a contrastive loss ${ℒ}_{con}$ that enhances the separability of style features through positive and negative pair constraints, forming clear intra-class compactness and inter-class dispersion in the embedding space. Meanwhile, the causal counterfactual attention module introduces a causal reinforcement loss ${ℒ}_{causal}$. By measuring the prediction deviation between factual attention and counterfactual attention, it guides the model to strengthen style regions with true causal contribution and suppress spurious activations induced by background or subject shape, thereby improving the causal rationality of attention learning.

Finally, the overall training objective is constructed as a weighted combination of the above losses:


$$\begin{array}{c} ℒ={ℒ}_{cls}+\lambda_{1}{ℒ}_{con}+\lambda_{2}{ℒ}_{causal} \end{array}$$ (17)

where $\lambda_{1}$ and $\lambda_{2}$ control the weights of the style contrastive constraint and the causal reinforcement constraint, respectively.

## 3. Experimental results

### 3.1. Datasets

To evaluate the effectiveness of the proposed MCS-Net for art style classification, we conduct experiments on three public and widely used painting datasets, namely WikiArt, MultitaskPainting100k, and Pandora18k. These datasets are collected from real artworks and cover a wide range of styles from classical to modern, including different art movements and works by diverse artists. Style labels are provided through manual or semi-automatic annotation and have been frequently used in existing studies on art style analysis and classification. The numbers of classes and the sample sizes of the training, validation, and test sets are summarized in Table 1.

**Table 1:** Statistics of the experimental datasets.

Dataset	Class	Training	Testing	Validation
WikiArt	27	57011	8144	16289
MultitaskPainting100k	125	69871	9982	19963
Pandora18k	18	12626	1804	3608

The WikiArt dataset is collected from the online WikiArt art platform and contains paintings from different movements and artists. It is annotated according to commonly used art-historical style categories and covers multiple representative art styles. Different styles show clear differences in color usage, brushstroke patterns, and compositional structure.

MultitaskPainting100k is a large-scale painting dataset with multiple art-attribute annotations, where artistic style is one of the core annotation tasks. This dataset contains a larger number of style categories and covers many fine-grained styles. It is therefore often used to evaluate a model’s recognition ability under a large number of classes and complex style distributions. Because different styles exhibit substantial overlap in visual appearance, this dataset poses higher requirements on discriminative learning.

Pandora18k is a medium-scale art style dataset that includes several common style categories. It is commonly used in prior work for art style classification and related analysis tasks. Although the dataset is relatively small, its style labels are relatively clear, making it suitable for validating model stability and generalization under limited data conditions.

### 3.2. Evaluation metrics

To comprehensively evaluate the performance of MCS-Net in the task of artistic style classification, we adopt Accuracy (Acc), Precision (Pre), Recall (Re), and F1-score (F1) as evaluation metrics. These metrics measure classification performance from different perspectives and can more fully reflect the overall performance of the model in multi-class artistic style recognition scenarios.

In the test set, for a given class, let the numbers of true positives, true negatives, false positives, and false negatives be *TP*, *TN*, *FP*, and *FN*, respectively. Then Accuracy, Precision, Recall, and F1-score are defined as follows:


$$\begin{array}{c} Acc=\frac{TP+TN}{TP+TN+FP+FN} \end{array}$$ (18)

$$\begin{array}{c} Pre=\frac{TP}{TP+FP} \end{array}$$ (19)

$$\begin{array}{c} Re=\frac{TP}{TP+FN} \end{array}$$ (20)

$$\begin{array}{c} F1=\frac{2\cdot Pre\cdot Re}{Pre+Re} \end{array}$$ (21)

### 3.3. Experimental settings

All experiments are implemented using the PyTorch deep learning framework and conducted on an NVIDIA RTX 3090 GPU. We adopt ResNet-101 pretrained on ImageNet as the backbone to extract high-level representations of artistic images. All input images are resized to 256×256 and then cropped to 224×224 as network inputs. During training, we apply random cropping and horizontal flipping, and we further introduce random rotation to enhance the model’s adaptability to spatial structural variations in artworks. During testing, we use center cropping to ensure deterministic evaluation. The model is trained end to end for 100 epochs with a batch size of 32, and optimization is performed using stochastic gradient descent with a momentum of 0.9. To mitigate overfitting, we apply weight decay with a coefficient of $1\times 10^{-5}$. The initial learning rate is set to $1\times 10^{-3}$, and a fixed-period learning rate decay strategy is used, where the learning rate is multiplied by 0.9 every 2 epochs. To improve reproducibility, the main hyperparameter settings of MCS-Net are summarized in Table 2, including both optimization-related configurations and key model-specific parameters used in the proposed modules.

**Table 2:** Main hyperparameter settings of MCS-Net.

Hyperparameter	Description	Value
Input size	Input image resolution	224×224
Backbone	Feature extraction backbone	ResNet-101
Optimizer	Optimization method	SGD
Momentum	Momentum coefficient	0.9
Weight decay	Weight decay coefficient	$1\times 10^{-5}$
Initial learning rate	Initial learning rate	$1\times 10^{-3}$
Batch size	Training batch size	32
Epochs	Number of training epochs	100
*L*	Number of attention maps	32
γ	Perturbation strength in Eq. (2)	0.1
$\lambda_{c}$	Channel dependency weight in Eq. (6)	0.5
$\lambda_{s}$	Spatial dependency weight in Eq. (6)	0.5
τ	Temperature parameter in contrastive loss	0.07
$\lambda_{1}$	Weight of contrastive loss in Eq. (17)	0.8
$\lambda_{2}$	Weight of causal loss in Eq. (17)	0.6

### 3.4. Comparison with other methods

#### 3.4.1. Performance comparison.

To evaluate the effectiveness of our proposed MCS-Net, we compare it with convolutional neural network models (ResNet50, ResNet101, ConvNeXt), attention- or structure-enhanced models (CAL [34], GOYA [35], DSGM [24]), and Transformer models and their variants (ViT, FAL-ViT [36], ACC-ViT [37]) on three public datasets, WikiArt, MultitaskPainting100k, and Pandora18k. We report classification accuracy, precision, recall, and F1. The results are summarized in Table 3, 4 and Table 5.

**Table 3:** Performance comparison of different methods on the WikiArt dataset.

Model	Backbone	Acc(%)	Pre(%)	Re(%)	F1(%)
ResNet50	ResNet-50	63.78	63.32	63.13	62.74
ResNet101	ResNet-101	64.11	64.30	63.04	63.13
CAL [34]	ResNet-101	67.57	68.88	65.54	66.81
ConvNeXt	ConvNeXt-Base	65.75	67.64	65.25	65.54
ViT	ViT-B_16	67.00	66.65	66.80	65.89
FAL-ViT [36]	ViT-B_16	67.24	67.15	66.85	66.24
ACC-ViT [37]	ViT-B_16	67.14	67.35	67.05	66.27
DSGM [24]	ConvNeXt-Base	66.35	66.44	65.26	65.01
GOYA [35]	ResNet-50	64.68	65.59	64.79	64.26
MCS-Net	ResNet-101	68.62	69.40	72.32	70.17

**Table 4:** Performance comparison of different methods on the MultitaskPainting100k dataset.

Model	Backbone	Acc(%)	Pre(%)	Re(%)	F1(%)
ResNet50	ResNet-50	57.65	49.20	58.00	51.27
ResNet101	ResNet-101	58.54	50.88	59.55	52.99
CAL [34]	ResNet-101	62.14	58.22	64.29	59.54
ConvNeXt	ConvNeXt-Base	61.27	57.40	63.29	58.52
ViT	ViT-B_16	62.13	58.15	63.86	59.24
FAL-ViT [36]	ViT-B_16	62.26	58.26	64.35	59.58
ACC-ViT [37]	ViT-B_16	62.58	58.57	63.87	59.60
DSGM [24]	ConvNeXt-Base	61.98	58.08	63.76	59.15
GOYA [35]	ResNet-50	58.09	50.32	58.78	52.37
MCS-Net	ResNet-101	64.07	60.69	65.83	61.87

**Table 5:** Performance comparison of different methods on the Pandora18k dataset.

Model	Backbone	Acc(%)	Pre(%)	Re(%)	F1(%)
ResNet50	ResNet-50	65.48	66.19	65.88	65.76
ResNet101	ResNet-101	67.59	68.05	68.07	67.81
CAL [34]	ResNet-101	69.49	69.91	70.43	69.99
ConvNeXt	ConvNeXt-Base	67.23	67.82	67.77	67.57
ViT	ViT-B_16	70.04	70.90	70.86	70.60
FAL-ViT [36]	ViT-B_16	70.92	71.73	71.68	71.46
ACC-ViT [37]	ViT-B_16	70.70	71.40	71.45	71.18
DSGM [24]	ConvNeXt-Base	68.11	68.75	68.77	68.48
GOYA [35]	ResNet-50	66.03	66.67	66.39	66.29
MCS-Net	ResNet-101	71.85	72.63	72.69	72.41

Overall, the comparison results across the three datasets indicate that different model families exhibit different strengths in art style classification. CNN-based methods provide effective local representation learning, while Transformer-based methods benefit from stronger global dependency modeling. However, when style evidence is distributed across multiple regions and mixed with content-related interference, relying mainly on either global representation or stronger backbones may still be insufficient. In comparison, the proposed MCS-Net achieves more consistent performance across datasets with different category numbers and style complexities, which suggests that jointly modeling global structure, complementary local regions, and refined attention responses is beneficial for robust style recognition.

Table 3 shows that MCS-Net achieves 68.62% accuracy and a 70.17% F1 score on WikiArt, yielding the best overall performance. Among CNN baselines, ResNet101 reaches 64.11% accuracy, indicating that relying primarily on a local texture-oriented inductive bias leaves room for improvement when style evidence is spatially dispersed and content varies substantially. The attention-enhanced method CAL obtains 67.57% accuracy, suggesting that improved attention selection can increase responses to informative regions and outperform conventional CNNs, yet it is still difficult to stably cover diverse style-discriminative cues. On the Transformer side, ACC-ViT reaches 67.14% accuracy, reflecting the advantage of global self-attention in modeling compositional relations, while remaining limited in aggregating fine-grained brushstroke and texture evidence. In comparison, MCS-Net improves accuracy over CAL and ACC-ViT and also achieves a higher F1 score, indicating more effective integration of multi-region discriminative evidence and better joint control of false positives and false negatives.

Table 4 shows that on MultitaskPainting100k with a larger number of categories, MCS-Net reaches 64.07% accuracy and a 61.87% F1 score, where the advantage becomes more pronounced. Compared with the representative CNN baseline ResNet101, which yields 58.54% accuracy and a 52.99% F1 score, the results suggest that under many classes and fine-grained differences, a single global representation is more likely to suffer from insufficient inter-class separation. The structure and relation modeling method GOYA achieves 58.09% accuracy, indicating that structured modeling can help capture local differences, but it remains inadequate when style boundaries are ambiguous. The Transformer variant FAL-ViT achieves 62.26% accuracy, implying that global modeling alone benefits from stronger discriminative constraints for fine-grained style distinction. Relative to ResNet101, MCS-Net improves both accuracy and F1 score, and it also improves accuracy over FAL-ViT. These results demonstrate that MCS-Net more effectively enhances feature separability in large-category scenarios, leading to stable gains in overall performance.

Table 5 shows that MCS-Net achieves 71.85% accuracy and a 72.41% F1 score on Pandora18k, maintaining the best overall performance. This dataset contains both local texture and global composition differences. The CNN backbone ConvNeXt achieves 67.23% accuracy, suggesting that strengthening local representations can improve results, but integrating cross-region style evidence remains limited. The structure-enhanced method DSGM reaches 68.11% accuracy, indicating that relation enhancement alone does not consistently yield strong overall improvement. The Transformer variant ACC-ViT achieves 70.70% accuracy, showing that global self-attention provides clear benefits on this dataset. Compared with ACC-ViT, MCS-Net improves accuracy, suggesting that while preserving both local style textures and global structural information, it can further enhance overall discriminability and stability.

#### 3.4.2. Statistical significance analysis.

To further verify whether the performance improvements of MCS-Net are robust rather than caused by random initialization, we repeated the experiments over five runs with different random seeds and performed paired t-tests between MCS-Net and the ResNet101 baseline. The results are reported in Table 6 as mean accuracy with standard deviation.

**Table 6:** Statistical significance analysis over five runs with different random seeds.

Dataset	Model	Accuracy (%)	Improvement (%)	p-value
WikiArt	ResNet101	64.03±0.10	–	–
	MCS-Net	68.62±0.11	4.59	$5.62\times 10^{-9}$
MultitaskPainting100k	ResNet101	58.46±0.10	–	–
	MCS-Net	64.10±0.11	5.64	$5.35\times 10^{-9}$
Pandora18k	ResNet101	67.60±0.11	–	–
	MCS-Net	71.92±0.10	4.32	$5.60\times 10^{-9}$

As shown in Table 6, MCS-Net consistently achieves higher mean accuracy than ResNet101 on all three datasets. On WikiArt, MCS-Net obtains an accuracy of 68.62±0.11%, compared with 64.03±0.10% for ResNet101, corresponding to an average improvement of 4.59%. On MultitaskPainting100k, MCS-Net achieves 64.10±0.11%, improving over ResNet101 by 5.64%. On Pandora18k, MCS-Net reaches 71.92±0.10%, which is 4.32% higher than ResNet101. The paired t-test results further show that the improvements are statistically significant, with p-values of $5.62\times 10^{-9}$, $5.35\times 10^{-9}$, and $5.60\times 10^{-9}$ on WikiArt, MultitaskPainting100k, and Pandora18k, respectively. These results indicate that the reported gains are stable across different random seeds and are unlikely to be artifacts of network initialization.

### 3.5. Comparison with other methods

#### 3.5.1. Effectiveness of the proposed components.

To verify the effectiveness of each component, we conducted ablation experiments by progressively introducing the basic style feature encoding module (BSFEM), the attention generation module (AGM), the style contrastive learning module (SCLM), and the causal counterfactual attention module (CCAM) on top of the ResNet101 backbone, and the results are reported in Table 7. Overall, the classification accuracy on all three datasets increases monotonically as more modules are added, indicating that each component provides a stable gain. The basic style feature encoding module yields a consistent relative improvement over ResNet101 across the three datasets, suggesting that strengthening style characteristics on top of convolutional representations improves discriminability. After further adding the attention generation module, the accuracy improves by 1.06%–1.76% relative to the previous stage, showing that multi region attention helps cover complementary style evidence and reduces interference from irrelevant regions. With the style contrastive learning module added, the accuracy further increases by 1.35%–1.71%, indicating that contrastive constraints enhance inter class separability and alleviate confusion among similar styles. Finally, introducing the causal counterfactual attention module brings an additional improvement of 1.08%–1.18%, demonstrating that constraining the true contribution of attended regions suppresses spurious responses and improves decision stability.

**Table 7:** Ablation results of different module combinations in terms of accuracy (%).

BSFEM	AGM	SCLM	CCAM	WikiArt	MultitaskPainting100k	Pandora18k
√				64.91	59.42	68.19
√	√			66.12	61.18	69.25
√	√	√		67.47	62.89	70.77
√	√	√	√	68.62	64.07	71.85

#### 3.5.2. Number of attention maps.

To further investigate the impact of the number of attention maps in the attention generation module on the recognition performance of MCS-Net, we evaluated *L* = 4, 8, 16, 32, and 64 on the three datasets, and the results are shown in Table 8. As *L* increases from 4 to 32, the classification accuracy consistently improves on all three datasets, and on WikiArt it increases from 67.84% to 68.62%. This trend indicates that using an appropriate number of attention maps improves the coverage of multi region style evidence, enabling the model to better capture style cues such as brushstroke textures, color layering, and compositional rhythm across different spatial locations and scales. Meanwhile, too few attention maps lead to insufficient region representation, whereas increasing *L* further to 64 causes an accuracy drop, suggesting that too many attention maps may introduce redundant regions and increase overlap among regions, thereby weakening the concentration of effective discriminative information. Considering both performance and stability, we set *L* = 32 as the default in subsequent experiments.

**Table 8:** Effect of the number of attention maps on the accuracy (%) of MCS-Net.

Number of attention maps *L*	WikiArt	MultitaskPainting100k	Pandora18k
4	67.84	63.45	71.08
8	67.91	63.49	71.13
16	68.06	63.62	71.27
32	68.62	64.07	71.85
64	68.26	63.79	71.34

#### 3.5.3. Balancing parameters $\lambda_{1}$ and$\;\lambda_{2}$.

**Fig 2:**
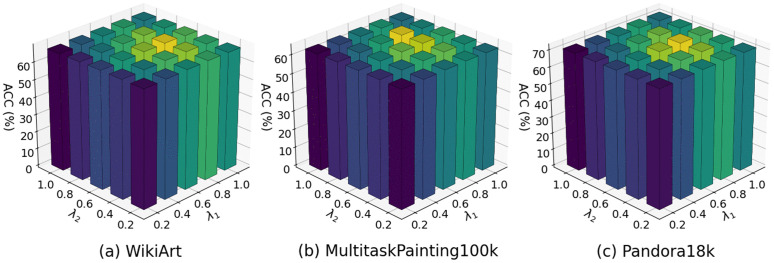
Impact of different balancing parameters on the classification accuracy of MCS-Net.

To analyze how the balancing parameters in the loss function affect training and performance, we performed a grid search over multiple candidate combinations, and the classification accuracy results are shown in Fig 2. The overall trend suggests that the model more readily achieves better performance on all three datasets when $\lambda_{1}$ and $\lambda_{2}$ are assigned moderately large weights. Considering the results across different combinations, the model achieves 68.62% on WikiArt, 64.07% on MultitaskPainting100k, and 71.85% on Pandora18k, which provides the most stable and near optimal overall performance; therefore, we use this setting as the default balancing parameter configuration in subsequent experiments.

### 3.6. Visualization and analysis

**Fig 3:**
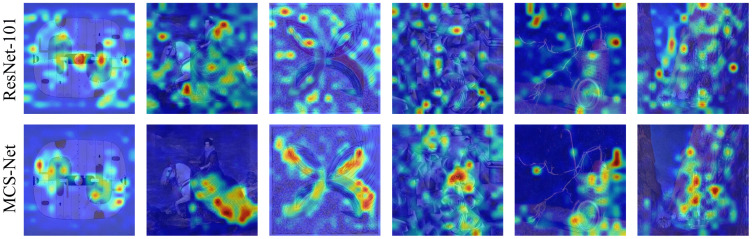
Visualization of attention responses for different methods.

To provide intuitive evidence on the spatial responses learned by MCS-Net, we visualize the attention maps on representative samples from the WikiArt dataset, as shown in Fig 3. Compared with ResNet101, MCS-Net produces responses that are more concentrated on regions closely related to style cues, especially areas containing brushstroke textures, color transitions, and structural boundaries. In contrast, the baseline model tends to exhibit more diffuse activation over broader image regions. These observations suggest that the proposed multi-region attention mechanism is more effective at capturing complementary style-related evidence while reducing interference from non-style content. Although such visualization does not by itself constitute a complete explanation of model decisions, it provides qualitative support for the claim that the proposed attention and counterfactual design helps the model focus on regions with stronger discriminative contribution.

### 3.7. Model complexity analysis

**Fig 4:**
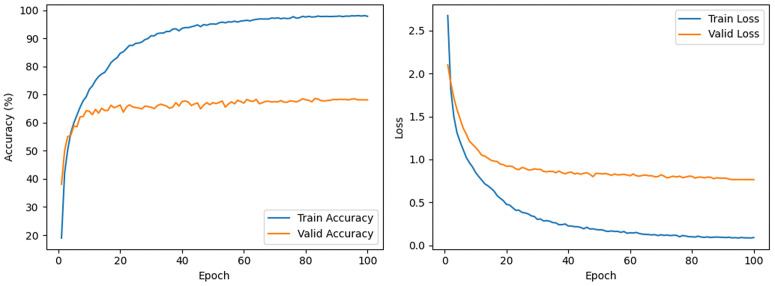
Training and validation curves of MCS-Net on the WikiArt dataset.

Fig 4 shows the changes in training and validation accuracy and loss of MCS-Net on the WikiArt dataset, which reflect the optimization process and convergence behavior during training. From the accuracy curves, the model converges quickly at the early stage. At around epoch 10, the training accuracy reaches about 73.3% and the validation accuracy reaches about 64.5%. The growth then slows down, and the validation accuracy increases to 67.95% at around epoch 50. From the loss curves, the training loss continuously decreases from about 2.7 and converges to about 0.2 in the later stage, while the validation loss decreases from about 2.1 to about 0.9 and the decline becomes smaller in the second half of training. Overall, both accuracy and loss exhibit a typical pattern of rapid change followed by stable convergence. The validation curves remain stable in the middle and late stages without sustained reverse fluctuations, indicating a stable training process and good generalization performance.

To evaluate the deployment potential of the model, we compare representative methods on the WikiArt dataset in terms of the number of parameters (Params) and floating point operations (FLOPs), and the results are reported in Table 9. MCS-Net achieves an accuracy of 68.62% with 43.75M parameters and 9.89G FLOPs, showing a good performance efficiency trade off. Compared with ResNet101, which has 42.54M parameters and 7.85G FLOPs, MCS-Net has a similar model scale but reaches higher accuracy, indicating that the improvement is not obtained by substantially increasing model capacity. Compared with the attention enhanced CNN method CAL, which has 43.68M parameters and 10.01G FLOPs and thus a similar complexity level, its accuracy is 67.57%, which is lower than MCS-Net, suggesting that our method yields a larger effective gain under comparable computational cost. In addition, Transformer based methods are notably heavier. ViT uses 85.66M parameters and 16.87G FLOPs, ACC-ViT uses 92.79M parameters and 17.16G FLOPs, and FAL-ViT has 83.99G FLOPs, which leads to a much higher computational cost.

**Table 9:** Efficiency comparison of different methods on the WikiArt dataset.

Model	Acc(%)	Params(M)	FLOPs(G)
ResNet50	63.78	23.54	4.13
ResNet101	64.11	42.54	7.85
CAL	67.57	43.68	10.01
ViT	67.00	85.66	16.87
FAL-ViT	67.24	88.19	83.99
ACC-ViT	67.14	92.79	17.16
MCS-Net	68.62	43.75	9.89

## 4. Conclusion

We focus on key challenges in art style classification, including dispersed style elements, complex local textures, and strong background interference, and we propose a Multi-Source Collaborative Style Network, MCS-Net, which jointly models global composition and multi-region local style information within a unified framework. By mining complementary style-discriminative regions with the attention generation module, shaping a more discriminative embedding space with the style contrastive learning module, and optimizing attention distributions from the perspective of causal contribution with the causal counterfactual attention module, MCS-Net effectively suppresses spurious correlation responses caused by content and background while providing more interpretable attention behavior and counterfactual evidence for style-discriminative region analysis. Experimental results show that our method achieves better performance than multiple representative approaches on three public datasets, WikiArt, MultitaskPainting100k, and Pandora18k. In future work, we will investigate two directions: first, we will study more robust style supervision and noisy label handling strategies to mitigate the impact of ambiguous style boundaries and subjective annotations; second, we will explore cross-dataset and cross-domain generalization evaluation and adaptation methods to further improve stability and transferability under different media, imaging conditions, and artistic forms.
